# CSAlign and CSAlign-Dock: Structure alignment of ligands considering full flexibility and application to protein–ligand docking

**DOI:** 10.1016/j.csbj.2022.11.047

**Published:** 2022-11-26

**Authors:** Sohee Kwon, Chaok Seok

**Affiliations:** a Department of Chemistry, Seoul National University, Seoul 08826, South Korea; b Galux Inc, Seoul 08738, South Korea

**Keywords:** Structure alignment, Protein–ligand docking, Virtual screening, Structure-based drug discovery

## Abstract

Structure prediction of protein–ligand complexes, called protein–ligand docking, is a critical computational technique that can be used to understand the underlying principle behind the protein functions at the atomic level and to design new molecules regulating the functions. Protein-ligand docking methods have been employed in structure-based drug discovery for hit discovery and lead optimization. One of the important technical challenges in protein–ligand docking is to account for protein conformational changes induced by ligand binding. A small change such as a single side-chain rotation upon ligand binding can hinder accurate docking. Here we report an increase in docking performance achieved by structure alignment to known complex structures. First, a fully flexible compound-to-compound alignment method CSAlign is developed by global optimization of a shape score. Next, the alignment method is combined with a docking algorithm to dock a new ligand to a target protein when a reference protein–ligand complex structure is available. This alignment-based docking method, called CSAlign-Dock, showed superior performance to *ab initio* docking methods in cross-docking benchmark tests. Both CSAlign and CSAlign-Dock are freely available as a web server at https://galaxy.seoklab.org/csalign.

## Graphical abstract

**Figure:**
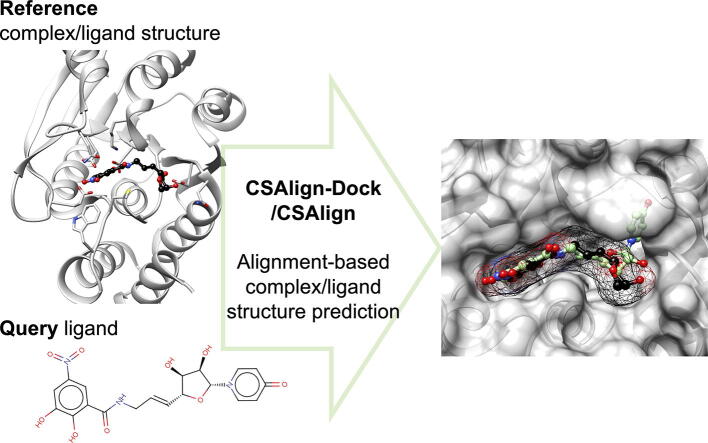

## 1 Introduction

A wide range of computational techniques has been used in drug discovery [1]. Recent advances in protein structure prediction by AlphaFold, [2] RoseTTAFold, [3] and related methods attract a lot of interest in computer-aided drug discovery (CADD) because accurate structure predictions are now available for protein targets with no experimentally determined structures. Structure-based approaches [4], [5] based on the three-dimensional protein structures are expected to be more promising than ever.

Despite the huge leap in protein structure prediction using deep learning [2], [3], [6], computational prediction of protein-compound complex structure, called protein–ligand docking, is still an unsolved problem [7], [8]. The docking accuracy of currently available programs highly depends on the input protein and ligand structures. The easiest docking type is “rigid self-docking” where the bound forms of both protein and ligand structures are given as input. One of the most popular docking types is “flexible self-docking” in which the bound protein structure and unbound ligand structure are provided as input. However, in many docking tests, unbound ligand structures are often generated by perturbing ligand torsions only, keeping bond lengths, angles, and ring puckers derived from the crystal structure. This leads to higher docking performance than using a truly unbound ligand structure.

Docking becomes more challenging in a realistic situation where the bound structures of protein and ligand are unknown [9]. One of the realistic docking types is cross-docking where a protein structure resolved in the unbound state or bound to a different ligand is used as input together with an unbound ligand structure. Model-docking in which a model protein structure is provided as input would be the most challenging type of docking that has to be explored by method developers more extensively. Docking difficulty depends also on the degree of conformational changes from the input protein structure, ranging from minor side-chain rotations to large backbone conformational changes [10].

Although the performance of *ab initio* docking dramatically decreases in realistic situations, it has been shown from previous studies that proper use of information on related protein–ligand interactions can be helpful. For example, interactions observed from a known complex structure with a different ligand can be used as positional restraints during docking or as a template to generate a binding pose by rigid alignment. The binding poses generated through rigid alignment often exhibit clashes between the protein and the ligand, which are removed by subsequent relaxation [11], [12], [13]. A different strategy uses an alignment-based score that compares the predicted pose and the known pose of a similar ligand to re-rank the poses generated by *ab initio* docking programs [14]. The alignment-based approaches can be further applied to virtual compound library screening [15] or to QSAR (quantitative structure–activity relationship) models [16].

In this work, we present two alignment methods, CSAlign and CSAign-Dock, that utilize the information of a known complex structure. CSAlign is a compound-to-compound structure alignment method that searches for the globally optimal alignment considering the full geometric degree of freedom of ligand including ring conformations, unlike other alignment methods which focus on fast alignment considering no or limited ligand flexibility [17], [18], [19], [20]. CSAlign-Dock performs docking by aligning to the reference ligand structure with additional consideration of protein–ligand interactions. Benchmark results show that CSAlign-Dock shows significantly higher success rates than *ab initio* docking methods in cross-docking benchmark tests.

Both CSAlign and CSAlign-Dock can be utilized in various ways for computer-aided drug discovery. Accurate compound structure alignment can be used for virtual screening tasks or QSAR. Accurate cross-docking can aid hit-to-lead optimization. CSAlign and CSAlign-Dock are freely available as a web server at https://galaxy.seoklab.org/csalign.

## 2 Methods

### 2.1 Overview of CSAlign and CSAlign-Dock protocol

**Fig. 1:**
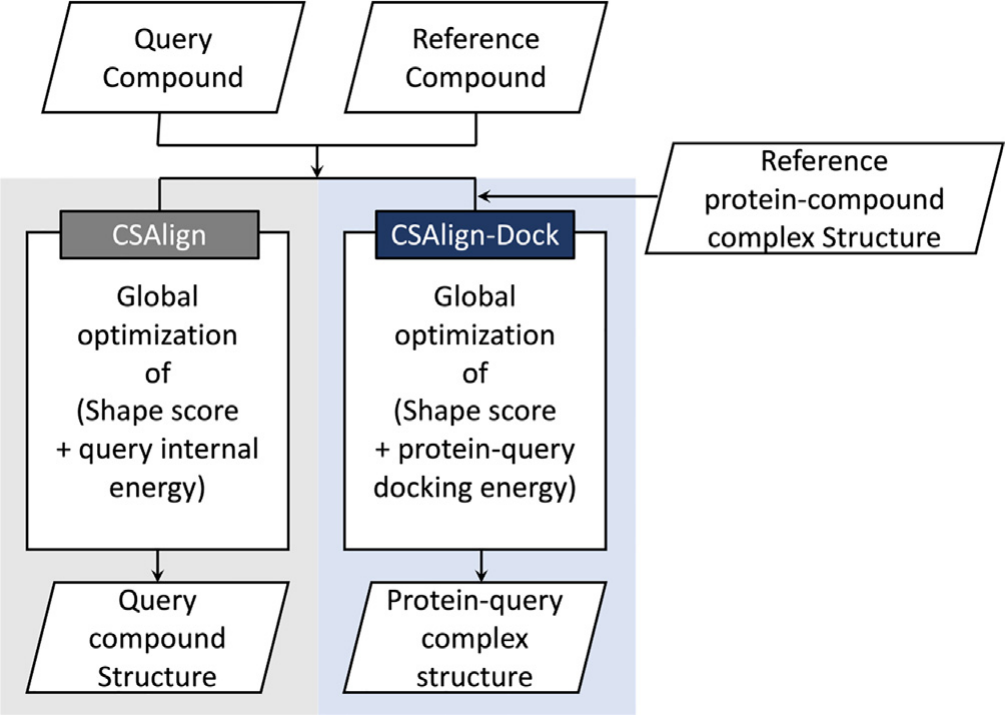
The overall procedure of CSAlign and CSAlign-Dock.

An outline of the small-molecule structure alignment program, CSAlign, and a protein–ligand docking program based on CSAlign, CSAlign-Dock, is depicted in Fig. 1. CSAlign takes a query compound and a 3D structure of a reference compound as input and returns the aligned compound structure. CSAlign-Dock takes a query compound and a reference protein–ligand complex structure as input and returns the protein–ligand complex structure for the query compound.

### 2.2 Scoring and sampling issues for CSAlign and CSAlign-Dock considering full compound flexibility

The difficulty in the alignment problem lies in both scoring and sampling. The scoring and sampling issues involved in CSAlign and CSAlign-Dock are briefly outlined here and described in detail in Subsections 2.2.1 and 2.2.2.

A score that defines the best-aligned conformation is not a naturally defined property, so an artificial function must be devised. Here, a simple shape match score is combined with an energy score to align two compounds (in CSAlign) or to align in the context of a protein-compound complex (in CSAlign-Dock). The scoring function is explained in detail in Subsection 2.2.1.

CSAlign and CSAlign-Dock sample full torsional, translational, and rotational degrees of freedom (DOF) of a query compound explicitly. Sampling ring conformations is not trivial because the geometric restraints for the cyclic structures must be satisfied. Here, ring conformations are sampled from the ring conformation library developed for GalaxyDock3 protein–ligand docking program [21]. The ring library consists of ring conformations collected from Cambridge Structural Database (CSD) [22] and BioLiP [23]. An average of 4.3 representative ring conformations per ring atom are deposited after hierarchical clustering with the maximum RMSD between two conformational cluster centers to 0.3√((ring size-1)/6) Å.

All DOFs are sampled simultaneously by a global optimization algorithm called conformational space annealing (CSA). CSA has been applied to various protein structure modeling problems such as protein–ligand docking and protein loop modeling [21], [24], [25], [26], [27], [28]. The CSA algorithm employed here is explained in detail in Subsection 2.2.2.

#### 2.2.1 Scoring function for evaluating aligned structures

To evaluate the alignment of the structure ($r_{Q}$) of the query compound Q to the structure ($r_{R}$) of the reference compound R, a shape match score $S_{shape}\left(r_{Q};r_{R}\right)$ is combined with an energy $E\left(r_{Q}\right)$ of the query compound as

(1) $$E_{align}\left(r_{Q};r_{R}\right)={S_{shape}\left(r_{Q};r_{R}\right)}^{-1\times sgn\;E(r_{Q})}\times E\left(r_{Q}\right)$$

where ‘sgn’ is the signum function. The shape score $S_{shape}$ ranges between 0 and 1 (better shape match if closer to 1), and the compound energy E has large negative values for favorable interactions and positive values for unfavorable interactions. The alignment energy $E_{align}$ is then large negative for a good shape match and favorable energy. This alignment energy is subject to global optimization to achieve the best structure alignment (with compound internal energy for E) and the best alignment docking (with docking energy for E).

The shape match score is defined as the sum of atomic match scores as $S_{shape}=\sum_{A\in Q,B\in R}V_{A,B}/max\left(\sum_{A\in Q}V_{A},\sum_{B\in R}V_{B}\right)$, where $V_{A}$ ($V_{B}$) is the volume of atom A (B) represented as a sphere of the van der Waals radius scaled down by 0.8, and $V_{A,B}$ is the volume overlapped between atoms A and B. The scale-down of the atomic radius is to emphasize core overlaps between atoms. The overlap volume $V_{A,B}$ is scaled down by 0.7 if the atom types of A and B are different.

For the energy *E*, the energy developed for the protein–ligand docking program GalaxyDock3 [21] is employed. The GalaxyDock3 energy is a hybrid of physics-based energy terms (Lennard-Jones energy $E_{vdw}$, hydrogen bond energy $E_{hb}$, Coulomb energy $E_{qq}$, and desolvation free energy $E_{desolv}$ of AutoDock4 [29] and modified ligand boned energy $E_{bonded}^{L}$ from CGenFF [30]), empirical energy (hydrophobic matching score $E_{HM}^{PL}$ of X-score [31]), and statistical energy (DrugScore $E_{DS}^{PL}$ [32]) defined as follows:

$$E_{GalaxyDock3}=E_{vdw}^{PL}+w_{1}E_{hb}^{PL}+w_{2}E_{qq}^{PL}+w_{3}E_{desolv}^{PL}+w_{9}E_{HM}^{PL}+w_{10}E_{DS}^{PL}+E^{L}$$

where

$$E^{L}=w_{4}E_{vdw}^{L}+w_{5}E_{hb}^{L}+w_{6}E_{qq}^{L}+w_{7}E_{desolv}^{L}+w_{8}\left(w_{4}E_{14-vdw}^{L}+w_{5}E_{14-hb}^{L}+w_{6}E_{14-qq}^{L}+w_{7}E_{14-desolv}^{L}\right){+w}_{11}E_{bonded}^{L}$$

The superscripts PL and L denote protein–ligand interaction energy and ligand internal energy, respectively, subscript 14 refers to 1–4 interaction energy often used in force field parametrization to scale down overestimated interactions between the atoms separated by three consecutive covalent bonds, and *w*_1_ to *w*_11_ are weight parameters for different energy terms that were optimized during the development process of GalaxyDock3. The energy *E* in Equation (1) used for CSAlign and CSAlign-Dock are $E^{L}$ and $E_{GalaxyDock3}$, respectively, as follows:

(2) $$E\left(r_{Q}\right)=\left\{\begin{array}{c} \;\;E^{L}\left(r_{Q}\right)\;\;\;\;for\;CSAlign\\ E_{\;GalaxyDock3}\left(r_{Q}\right)\;\;\;\;for\;CSAl\mathrm{ign}-Dock \end{array}\right)$$

#### 2.2.2 CSA global optimization for optimizing alignment

The CSA algorithm [21], [24], [25], [26], [27], [28] is used to find optimal alignment by global optimization of the alignment energy $E_{align}\left(r_{Q};r_{R}\right)$ defined in Equation (1) with respect to $\left\{r_{Q}\right\}$. CSA evolves a bank $\left\{r_{Q,i}\right\}$ of a fixed number (*i* = 1, …, 30) of conformations by generating new trial conformations and updating the bank iteratively. The final bank of 30 conformations is returned as output.

*Generating trial conformations and updating the bank*: Trial conformations are generated at each iteration step by crossover and mutation of the members of the current bank and initial bank. The distance among the bank members in the conformational space is controlled by a parameter called d-cut. Ligand RMSD is used as a distance measure. A trial conformation within d-cut from an existing bank conformation *i* replaces *i* if it has lower alignment energy than *i*. A trial conformation farther than d-cut from all bank members replaces the highest-energy bank conformation *j* if it has lower alignment energy than *j*. All bank and trial conformations are locally optimized before energy comparison. The iteration starts with diverse conformations and focuses on narrower areas of more optimal conformations by gradually reducing d-cut with iteration.

*Local optimization of a conformation*: Each conformation is optimized in translational, rotational, and torsional DOFs by local minimization of the alignment energy $E_{align}$.

*Generation of initial bank*: The initial bank not only provides the starting point of iteration but also a source of diversity when generating new trial conformations. A set of 30 structures are generated by randomly perturbing the query compound torsion angles and sampling ring conformations from the ring library with a Boltzmann probability [21]. Each conformation is then optimized by local minimization of the energy *E*, and five alignments are generated per conformation by a rigid alignment described in the next paragraph, resulting in 150 poses, which are reduced to 30 conformations by K-means clustering.

*Rigid alignment of a query conformation to the reference conformation*: A modified algorithm of PhaseShape [33] is employed, which rapidly overlays two atomic triads selected from the query and reference structure, respectively. An atom in the query structure is first paired with the most similar atom in the reference structure. The similarity between two atoms is defined as the cosine similarity between the radial distance distributions represented by the number of neighboring atoms within 6 Å binned in 1 Å. An additional penalty factor of 0.7 is multiplied for different atom types, unlike in the original algorithm [33]. All possible query-reference triad mappings are explored to minimize the sum of the squared Euclidean distance between the mapped atom pairs.

### 2.3 Test sets for performance comparison

The benchmark set for CSAlign-Dock was designed to test the alignment docking method in a cross-docking setting: docking of a compound to a protein structure other than its bound form. The PDBbind 2019 database [34] of 4,852 protein–ligand complex structures was processed by selecting complexes with proteins bound to multiple compounds and by removing redundancy in proteins as follows. First, the complex structures involving identical proteins were structurally aligned using TMalign [35], and the binding pockets were defined by hierarchical clustering of the ligand geometric centers with the RMSD cutoff of 10 Å. For proteins bound to multiple compounds at the same binding pocket, protein redundancy was removed by a sequence identity cutoff of 70 %. For each protein, a compound was randomly chosen as the query and the other compounds as references.

The final benchmark set consists of 477 query compounds (and the corresponding proteins) and 1,724 query-reference pairs of protein–ligand complexes. The 477 compounds were employed to evaluate the performance of CSAlign in self-alignment tasks relative to that of LS-align [17]. Cross-docking performance of CSAlign-Dock was benchmarked on the 1,724 cross-docking pairs (docking of the query compound to the protein structure bound to the reference compound) against other available docking programs. A list of PDB IDs for the benchmark set is available in Supplementary Table **ST 1**. The query compound structures were re-generated using CORINA [36] maintaining the stereochemistry at chiral centers. The compound structures thus have different bond lengths, angles, and ring conformations from the crystal structures. In the cases where CORINA failed to generate a structure with the desired stereochemistry, in-house sampling methods were used to generate the structures manually. The ligand structures were then minimized using OpenBabel [37]. UCSF Chimera [38] was used to add hydrogen atoms and to assign Gasteiger charges.

**Fig. 2:**
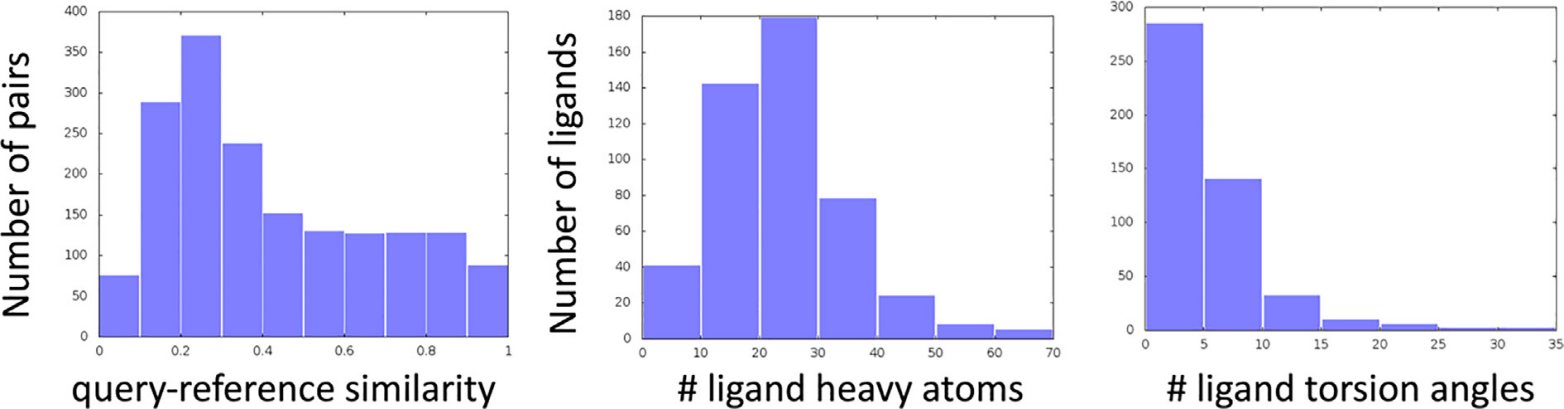
The ligand similarity distribution in the cross-docking benchmark set of 1,724 pairs (leftmost panel) and the ligand size distribution in the self-alignment test set of 477 compounds (two right panels).

Within the set of 1,724 query-reference pairs, each query compound has an average (median) of 3.6 (2.0) reference compounds with an average (median) similarity of 0.427 (0.347) measured by the Tanimoto coefficient calculated using OpenBabel [37] with default options. The distribution of the compound similarity in the set is shown in the leftmost panel of Fig. 2. The average (median) number of ligand heavy atoms and the number of flexible ligand torsion angles for the 477 query compounds are 24.4 (23.0) and 5.7 (5.0), respectively. The size distribution of the query compounds is shown in the two right panels of Fig. 2.

Another set denoted as the CDK2 set, collected by the authors of PhaseShape [33] was employed to compare the CSAlign and CSAlign-Dock performances with that of PhaseShape. The CDK2 set consists of 10 complex structures of CDK2 (Cyclin-dependent kinase 2) with its 10 inhibitors. A complete list of PDB IDs for the CDK2 set is available in Supplementary Table **ST2**. Following PhaseShape, the input ligand structures taken from the crystal complex structures were used after adding hydrogen atoms and assigning Gasteiger charges using UCSF Chimera. The average (median) number of heavy atoms and rotatable torsions are 27.2 (27.0) and 6.3 (5.5), respectively.

### 2.4 Running the comparing methods

The compound alignment program LS-align [17] is compared to CSAlign, and protein–ligand docking programs GalaxyDock3 [21] and AutoDockVina1.1.2 [39] are compared to CSAlign-Dock.

LS-align offers rigid alignment mode and flexible alignment mode. Here LS-align was run on the flexible alignment mode, Flexi-LS-align, which performs sampling of rotatable single bonds. Flexi-LS-align was run with two different options, default options and additional options, called “Flexi-LS-align (option)”, for more accurate alignment (with “md”, using more rotations of single bonds to generate more rotamers, and “acc”, taking more time to search for more alignments). Both Flexi-LS-align and “Flexi-LS-align (option)” return a single best-aligned compound structure as output.

GalaxyDock3 (https://galaxy.seoklab.org/softwares/galaxydock.html), a protein–ligand docking program that considers the full ligand flexibility, was run on default settings (bank size of 100, generating 100 complex structures within a cubic box of length 22.5 Å) using the geometric center of the reference compound as the binding center and considering cofactors within the binding pocket.

AutoDockVina1.1.2 [39] was also run on default settings with a cubic box of length 25 Å, generating an average of 8.8 conformations as the output on the cross-docking benchmark set.

## 3 Results and discussion

The performance of CSAlign and CSAlign-Dock was mainly tested on the benchmark set of 477 query compounds and 1,724 query-template compound pairs. The 477 query compounds were first used as a self-alignment benchmark set to test the performance of CSAlign in aligning a re-generated structure to the crystal pose. Then 1,724 compound pairs were used to evaluate CSAlign and CSAlign-Dock for the protein–ligand cross-docking problem.

### 3.1 Self-alignment performance of CSAlign

CSAlign was tested for self-alignment by aligning the newly generated query structures to the reference structures extracted from the crystal complex structures on the query compound set of 477 compounds. The aligned structures were evaluated by heavy atom RMSD to the corresponding reference structures considering the equivalence of symmetric ligand atoms. As shown in Table 1, CSAlign could align 96 % and 99 % of the query compounds within RMSD of 2.0 Å and 2.5 Å, respectively, showing higher performance than Flexi-LS-align and “Flexi-LS-align (option)”. The average RMSD was 0.61 Å, compared to 1.67 Å and 1.47 Å with Flexi-LS-align and “Flexi-LS-align (option)”, respectively. The cases where aligned compound shows RMSD higher than 2.5 Å include compounds with large macrocyclic or fused rings that the current ring library does not cover. While the LS-align methods focus on fast structure alignment for high-throughput virtual screening, CSAlign focuses on accurate alignment for effective ligand sampling.

**Table 1:** The performance comparison of CSAlign and Flexi-LS-align on a self-alignment benchmark set of 477 compounds in terms of the success rates (percentages of the cases in which RMSD of the highest-score alignment < 2 Å and < 2.5 Å) and the average RMSD of the best-scoring alignments.

**Alignment method**	**Success rate** **<2 Å (%)**	**Success rate** **<2.5 Å (%)**	**Average** **RMSD (Å)**
**CSAlign**	96	99	0.61
**Flexi-LS-align**	73	80	1.67
**Flexi-LS-align (option)**	77	83	1.47

**Fig. 3:**
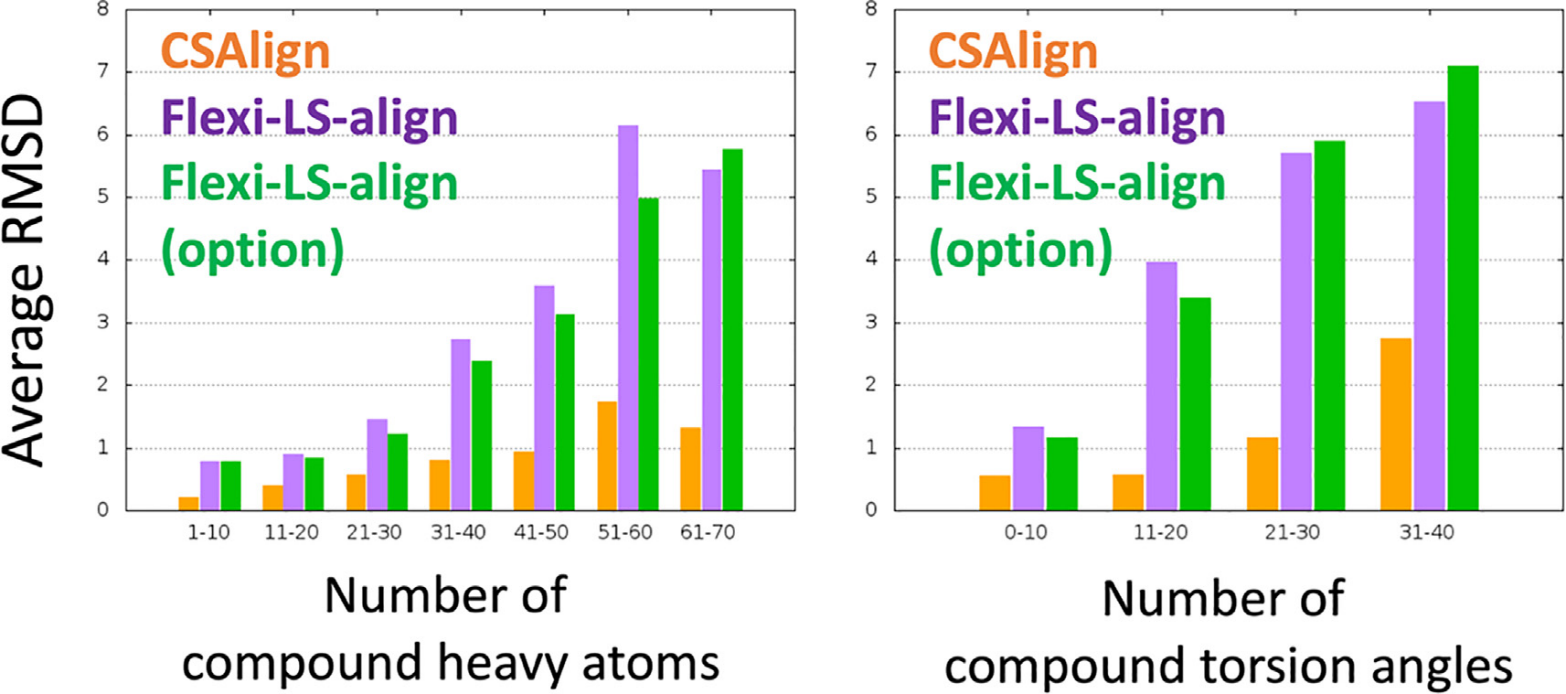
Average RMSD (Å) of the aligned structures to the crystal structures with respect to the number of compound heavy atoms and torsion angles for CSAlign, Flexi-LS-align, and Flexi-LS-align (option).

**Fig. 4:**
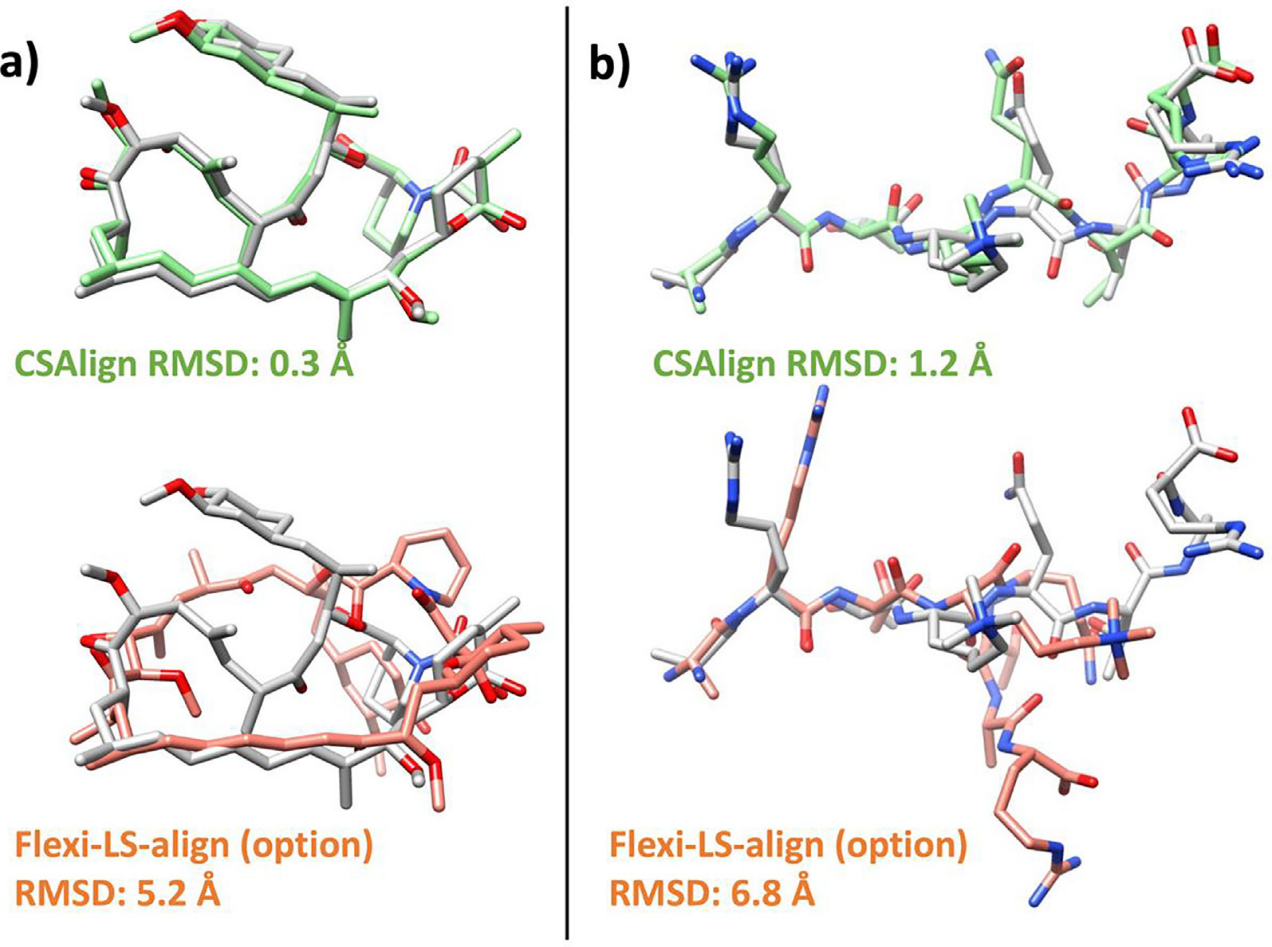
Self-alignment results for (a) Rapamycin immunosuppressant drug (PDB ID 1fkb) containing a macrocyclic ring of 29 backbone atoms and (b) Histone H3 (PDB ID 4 h75) with 35 torsion angles obtained by using CSAlign (green backbone structure) and “Flexi-LS-align (option)” (orange backbone structure). Crystal structures are colored in gray. (For interpretation of the references to color in this figure legend, the reader is referred to the web version of this article.)

RMSD increases for larger and more complex ligands, as shown in Fig. 3. Two examples in which CSAlign accurately aligns large and complex ligands compared to “Flexi-LS-align (option)” are illustrated in Fig. 4.

### 3.2 Cross-docking performance of CSAlign and CSAlign-Dock

When aligning a query compound to the reference structure, CSAlign considers ligand internal energy only while CSAlign-Dock considers protein–ligand interaction energy in addition. The effect of considering additional protein–ligand interaction energy was tested on the benchmark set of 1,724 cross-docking pairs in Subsection 3.2.1. The methods are also compared to *ab initio* protein–ligand docking methods that do not use the information on the reference structure in Subsection 3.2.2. In Subsection 3.2.3, the dependence of cross-docking performance on the query-reference similarity is analyzed.

#### 3.2.1 Cross-docking performance: Comparison between CSAlign and CSAlign-Dock

The performance of CSAlign and CSAlign-Dock on the cross-docking benchmark set of 1,724 pairs is summarized in Table 2 where success rates (percentages of the cases with predicted poses satisfying the RMSD criterion, < 2.5 Å or <2 Å from the crystal ligand structure) for the top 1 pose and the pose with the best RMSD are compared. CSAlign-Dock shows higher success rates than CSAlign for both top 1 and best poses.

**Table 2:** Success rates of CSAlign and CSAlign-Dock for the top1 pose and the pose with the best RMSD among 30 poses in the final bank on the cross-docking set.

**Performance measure**	**CSAlign**	**CSAlign-Dock**
	**Top 1 pose (Best pose)**	**Top 1 pose (Best pose)**
**Success rate < 2.5 Å (%)**	49 (63)	55 (73)
**Success rate < 2 Å (%)**	42 (56)	50 (68)

**Fig. 5:**
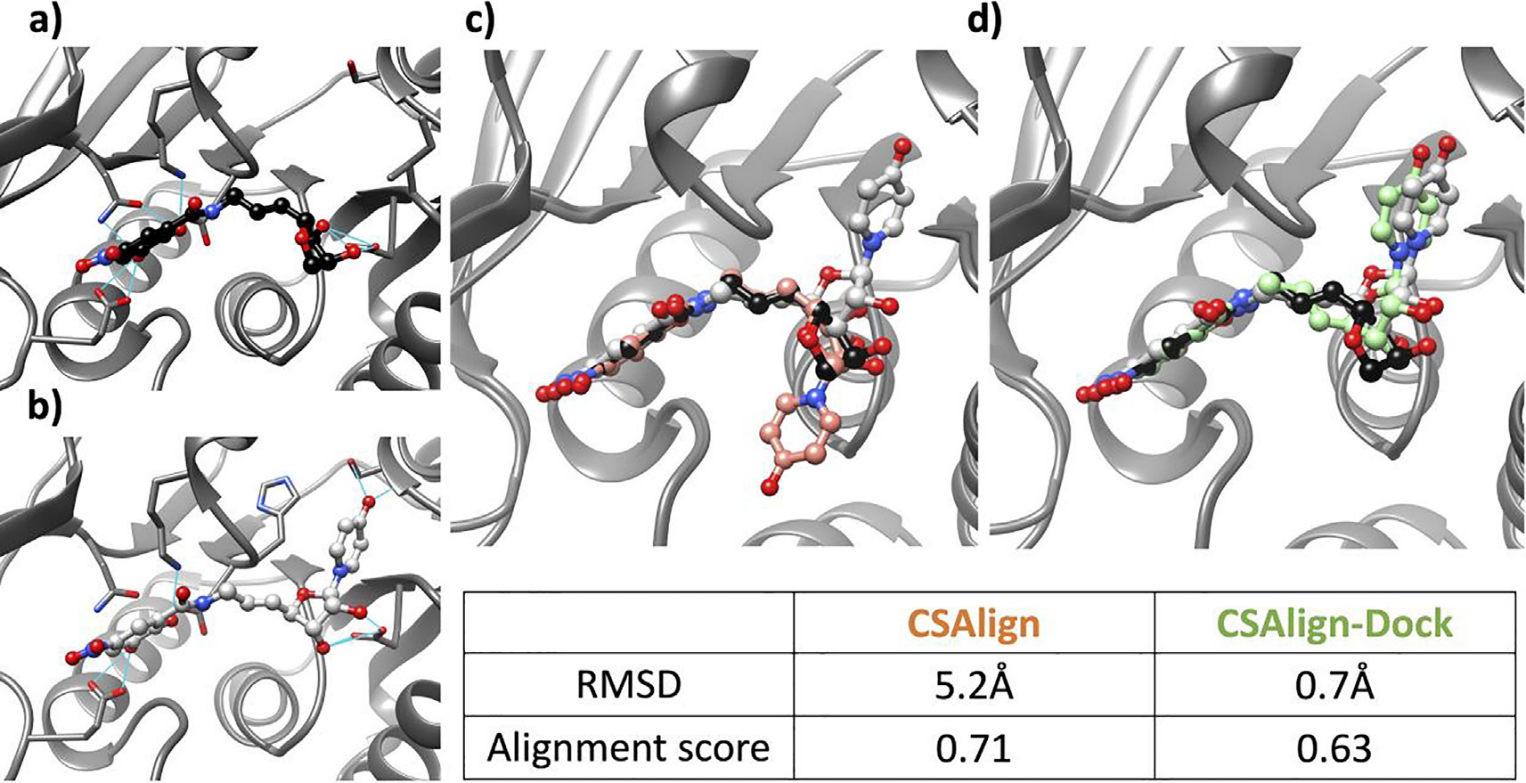
Comparison of CSAlign and CSAlign-Dock results on catechol O-methyltransferase (COMT). Comparison of (a) the crystal complex structure of COMT with the reference compound OZR (PDB ID 3ozr, crystal pose in black) and (b) that with the query compound OZZ (PDB ID 3ozt, crystal pose in gray) shows a part of the query compound uncovered by the reference compound. (c) The top 1 pose obtained by CSAlign (orange) does not locate the uncovered part of OZR correctly, (d) while CSAlign-Dock (green) does by additional consideration of protein–ligand interactions. (For interpretation of the references to color in this figure legend, the reader is referred to the web version of this article.)

Since CSAlign-Dock accounts for additional protein–ligand interaction energy, the method can help locate the parts of query compounds that the reference compounds do not cover. Fig. 5 shows such an example in which the ribose moiety of the query and the reference compounds are better matched by CSAlign (Fig. 5**c**), but CSAlign-Dock returns a conformation with more favorable protein–ligand interactions that show a different orientation at the anomeric center (Fig. 5**d**).

**Fig. 6:**
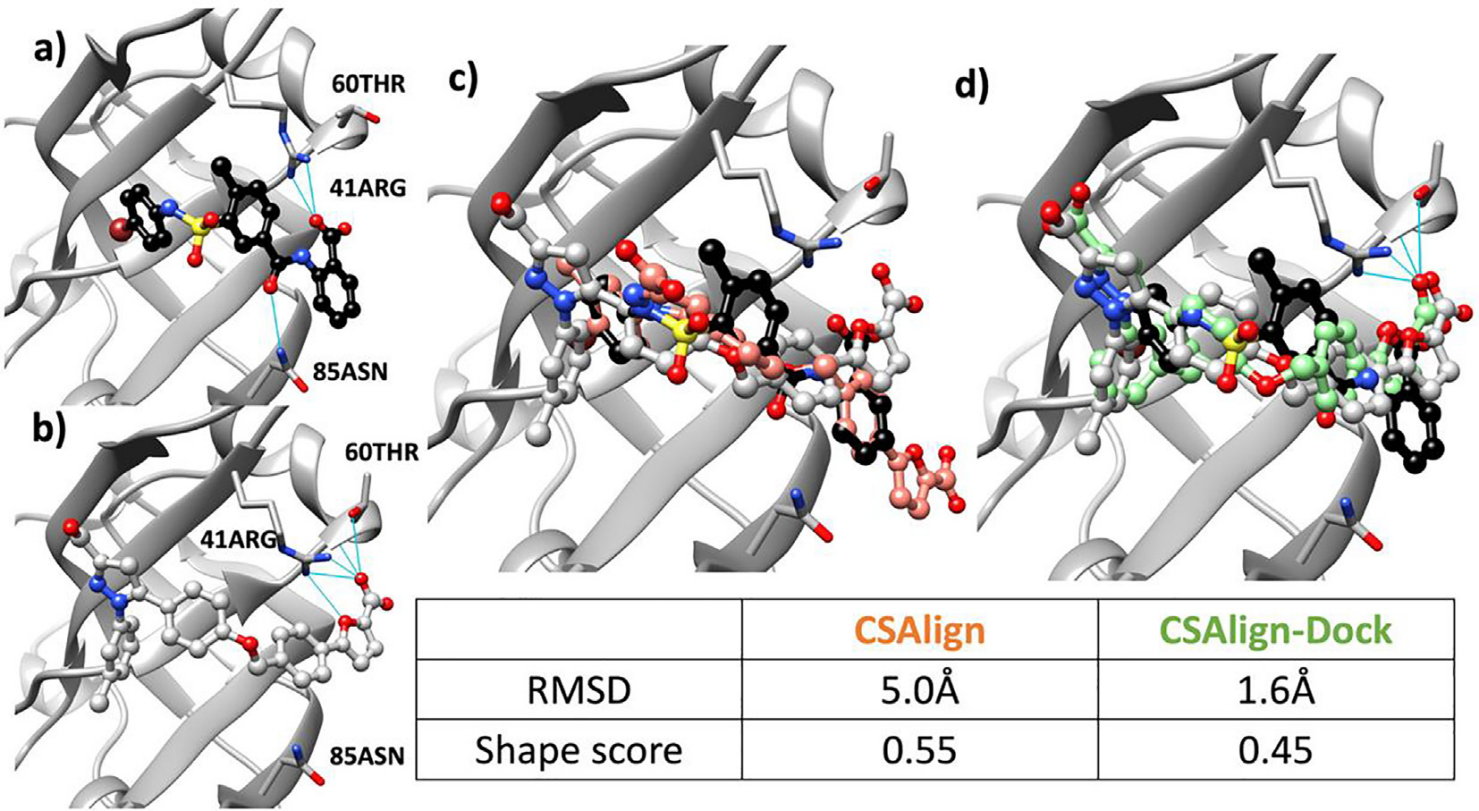
Comparison of CSAlign and CSAlign-Dock results on the *N*-terminal domain of the 70 kDa subunit of replication protein A (RPA70N). (a) RPA70N in complex with its inhibitor VU0085636 (PDB ID 5e7n, crystal pose colored in black) [40] and (b) potent inhibitor 1XT (PDB ID 4luz, crystal pose colored in gray) [41]. The top1 poses predicted using (c) CSAlign (orange) and (d) CSAlign-Dock (green) are compared. (For interpretation of the references to color in this figure legend, the reader is referred to the web version of this article.)

Fig. 6 illustrates how docking energy accounted for in CSAlign-Dock helps accurate pose prediction even for a query compound with low similarity to the reference (Tanimoto coefficient of 0.198). While the reference compound forms hydrogen bonds with 85ASN and 41ARG (Fig. 6**a**), the query compound interacts with 60THR instead of 85ASN (Fig. 6**b**). CSAlign-Dock was able to recover the different interaction patterns for the query compound, sacrificing the shape match.

#### 3.2.2 Cross-docking performance: Comparison to *ab initio* protein–ligand docking methods

Compared to *ab initio* docking programs, CSAlign-Dock uses additional information on a known complex structure of a reference compound both in sampling and scoring. The initial bank for CSAlign-Dock is generated by aligning to the reference compound, effectively reducing the conformational search space. Also, the objective function for optimization, the alignment energy, incorporates the score of shape match to the reference structure.

According to Table 3, the alignment-based docking CSAlign-Dock shows higher success rates in the cross-docking task (55 % for top 1 within <2.5 Å) compared to the two *ab initio* protein–ligand docking methods GalaxyDock3 and AutoDockVina (35 % and 33 %, respectively) on the cross-docking benchmark set of 1,724 pairs. GalaxyDock3 shows higher success rates than CSAlign-Dock when the best poses are considered because conformational sampling is not biased toward the reference structure. Success rates of *ab initio* docking to the bound crystal protein structures, called self-docking, performed with re-generated ligand structures were higher than the cross-docking success rates for both GalaxyDock3 and AutoDockVina, as shown in Table 3.

**Table 3:** Cross-docking success rates for CSAlign-Dock compared to cross-docking and self-docking success rates of two *ab initio* docking methods, GalaxyDock3 and AutoDockVina.

	Performance measure	CSAlign-Dock	GalaxyDock3	AutoDockVina
		**Top 1(Best)**	**Top 1(Best)**	**Top 1(Best)**
Cross-docking	**Success rate < 2.5 Å (%)**	55 (73)	35 (84)	33 (65)
	**Success rate < 2.0 Å (%)**	50 (68)	29 (78)	26 (55)
Self-docking	**Success rate < 2.5 Å (%)**	–	52 (89)	49 (74)
	**Success rate < 2.0 Å (%)**	–	48 (86)	40 (64)

**Fig. 7:**
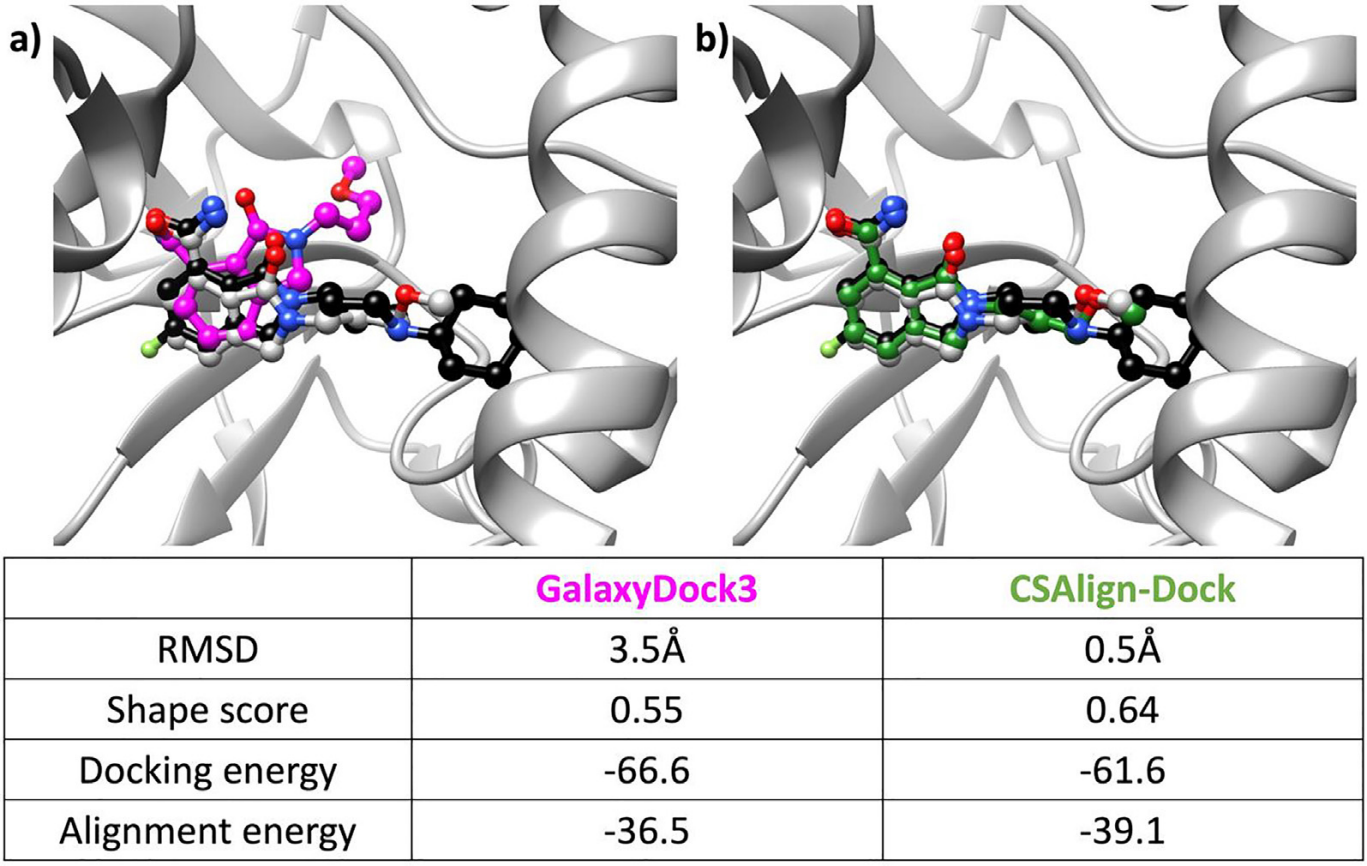
Cross-docking results of the query compound 10b (PDB ID 4zzx, crystal pose colored in gray) to the structure of PARP2 (Poly [ADP-ribose] polymerase) bound to the reference compound 20by bound (PDB ID 4zzy, crystal pose colored in black) using (a) GalaxyDock3 (predicted pose in pink) and (b) CSAlign-Dock (predicted pose in green). (For interpretation of the references to color in this figure legend, the reader is referred to the web version of this article.)

Fig. 7 shows an example where CSAlign-Dock outperforms *ab initio* docking by GalaxyDock3. In this case, GalaxyDock3 energy ranked a pose with RMSD 3.5 Å as top 1 even though a pose closer to the native state with RMSD 1.79 Å was sampled in the final bank (ranked 9th). On the other hand, CSAlign-Dock could sample a native-like conformation of RMSD 0.5 Å with the biased sampling and succeed in ranking the pose as top 1 with the biased score toward the reference. This example demonstrates that the alignment-based docking method CSAlign-Dock can predict complex structures more accurately than an *ab initio* docking method even when a dissimilar ligand is used as a template [12].

Both CSAlign-Dock and GalaxyDock3 were run with the default cubic box size of 22.5 Å. There are, however, large ligands in our benchmark set which require larger docking boxes. For such ligands, a larger box with the size of 30 Å was tried, but it did not lead to better docking results. This is because the larger search space for large ligands makes the docking problem more difficult. (See Supplementary Figure **SF1**).

#### 3.2.3 Cross-docking performance: Dependence on similarity to the reference

**Fig. 8:**
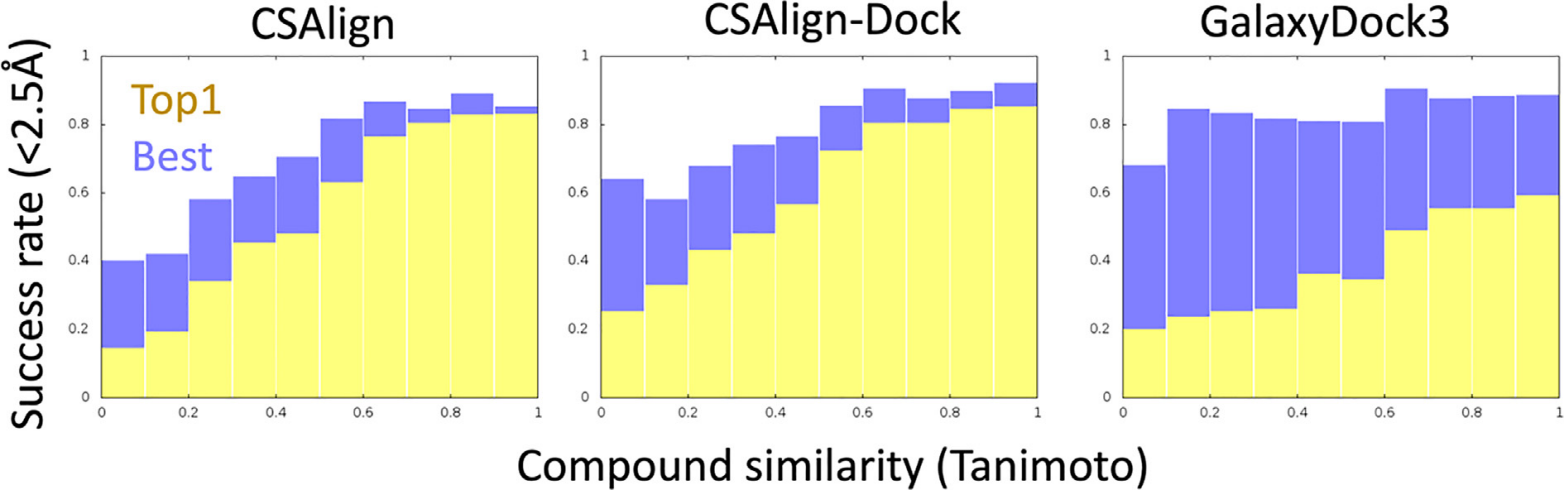
Dependence of success rates (with the RMSD cutoff of 2.5 Å) on compound similarity measured by the Tanimoto coefficient for CSAlign, CSAlign-Dock, and GalaxyDock3. The yellow bar shows the success rate of the top 1 pose and the blue bar the best pose. (For interpretation of the references to color in this figure legend, the reader is referred to the web version of this article.)

The alignment-based cross-docking results were also evaluated in terms of similarity between the query and the reference compound. Fig. 8 (Supplementary Figure **SF 2**) shows the dependence of the success rate (with the RMSD cutoff of 2.5 Å) on the 2D (3D) query-reference compound similarity for CSAlign, CSAlign-Dock, and GalaxyDock3. All three methods show an increased cross-docking success rate for more similar compounds. Such results are expected for CSAlign and CSAlign-Dock, which directly use the information on the reference structure both in sampling and scoring. The higher success rates of *ab initio* cross-docking for similar compounds are also expected because the protein structure is expected to involve smaller conformational changes for similar compounds. The same reasoning is applied to CSAlign-Dock, which considers protein–ligand interactions during docking. It is notable that alignment-based docking CSAlign-Dock performs better than both the pure alignment method and the *ab initio* docking method for dissimilar ligands (e.g., those with Tanimoto coefficient < 0.4). This result implies that CSAlign-Dock combines the advantages of information-based alignment and principle-based docking properly. This result is consistent with previous work [12].

### 3.3 Case study on the CDK2 set

**Fig. 9:**
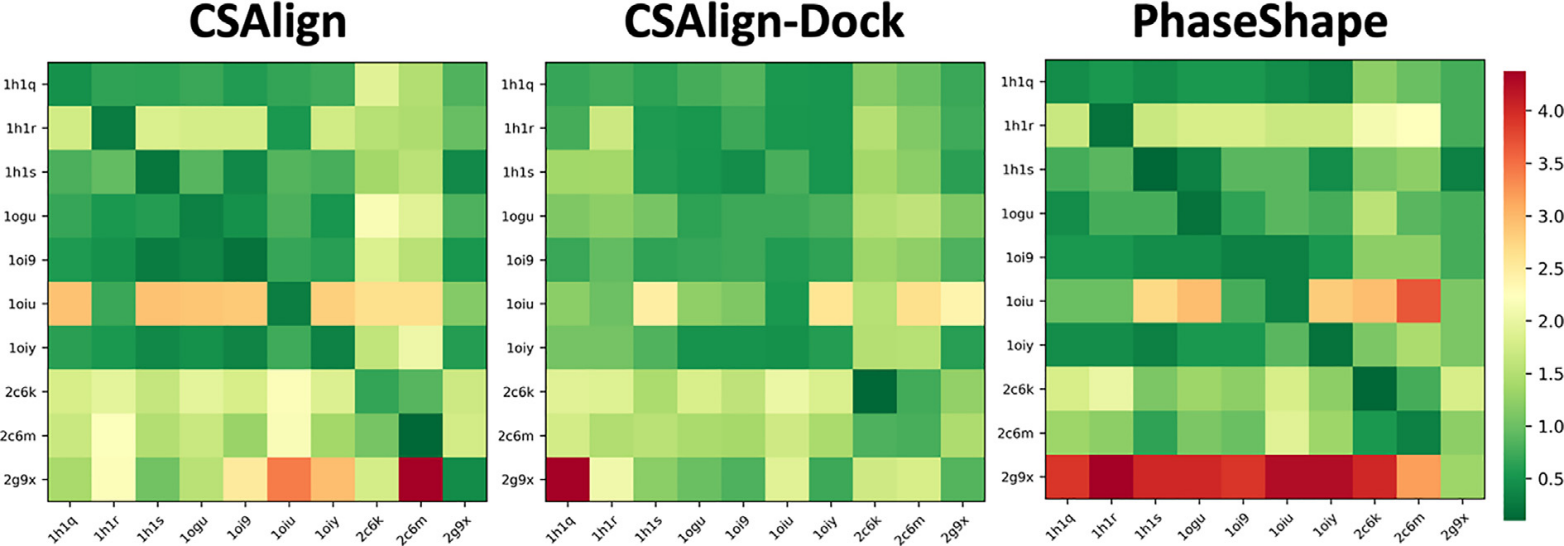
The 10 by 10 cross-docking results for the CDK2 set by CSAlign, CSAlign-Dock, and PhaseShape are shown. Each box is colored based on the top 1 RMSD, from green (low RMSD) to red (high RMSD) according to the color scale bar on the right. (For interpretation of the references to color in this figure legend, the reader is referred to the web version of this article.)

For the 10 complex structures in the CDK2 set, 10 by 10 cross-docking runs were carried out using CSAlign and CSAlign-Dock to compare with PhaseShape [33]. Fig. 9 shows that CSAlign-Dock shows the best performance with most predictions within RSMD < 2.0 Å (green color) from the crystal pose.

**Fig. 10:**
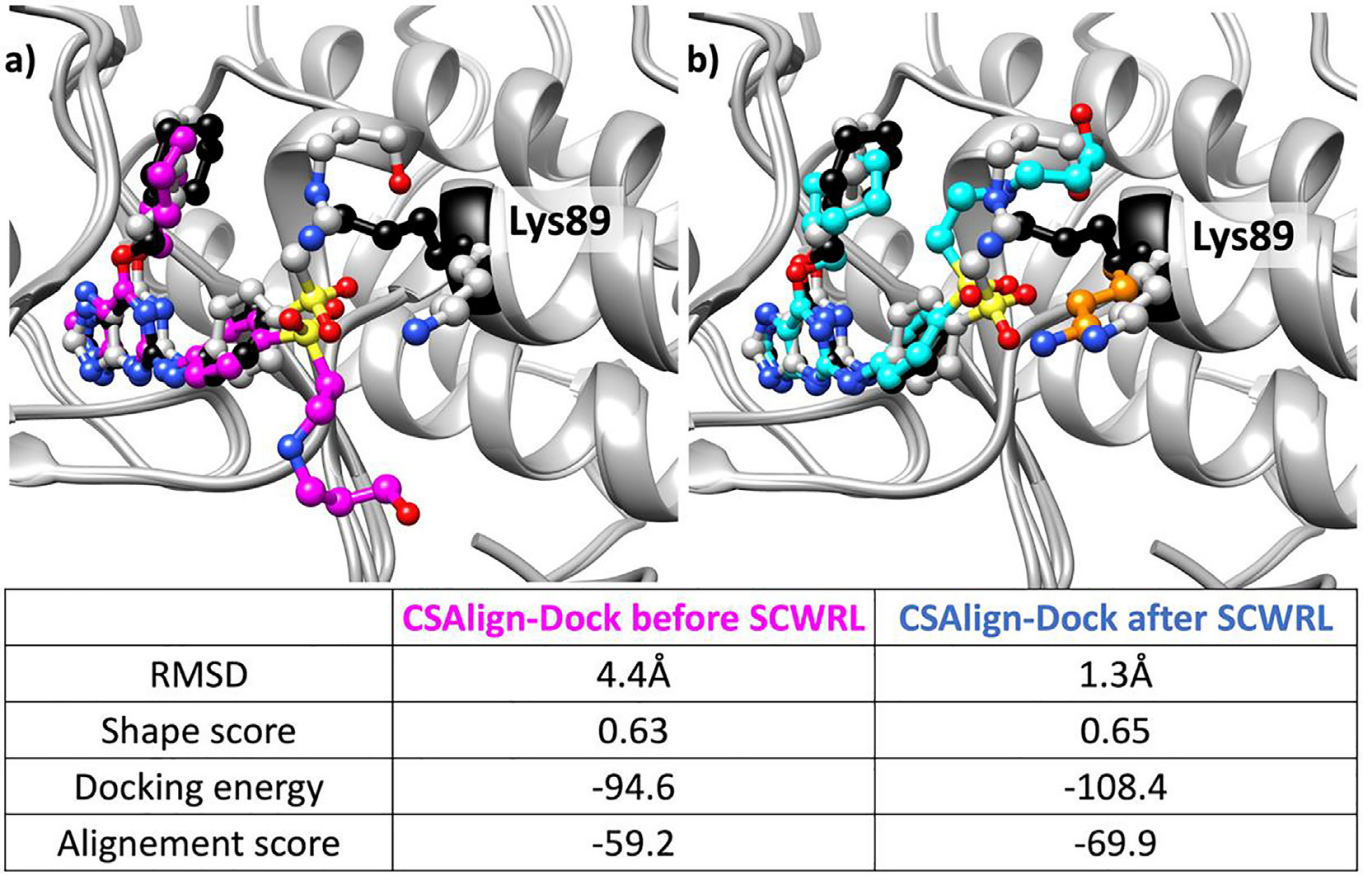
The top1 pose predicted by using CSAlign-Dock before and after protein sidechain repacking with SCWRL. The inhibitor NU6271 (PDB ID 2g9x, crystal pose colored in gray) was docked on the NU6094 bound CDK2 structure (PDB ID 1h1q, crystal pose colored in black). Figure (a) shows the docking results on the crystal protein structure (Lys 89 sidechain colored in black), and (b) shows the docking results on the protein structure after sidechain repacking using SCWRL (Lys 89 colored in orange). (For interpretation of the references to color in this figure legend, the reader is referred to the web version of this article.)

Compared to other inhibitors, docking of NU6271 (PDB ID 2g9x) shows the highest RMSD by PhaseShape and alignment methods. The high RMSD is due to the substituent in NU6271 connected to the sulfonyl group, which remains unaligned with the other inhibitors. CSAlign-Dock could locate the unaligned region of NU6271 except for cross-docking to NU6094 (PDB ID 1h1q). As shown in Fig. 10, in the CDK2 structure bound to NU6094, Lys89 blocks the pocket where NU6271 binds, unlike other CDK2 structures. As a case experiment, the whole sidechains of CDK2 were rebuilt using SCWRL [42] before docking with CSAlign-Dock. The top 1 pose using the rebuilt sidechains showed an RMSD of 1.3 Å with a better shape score and docking energy. This case implies that explicit consideration of protein conformational change could be an effective strategy for cross-docking with proper sampling and scoring [43].

## 4 Conclusions

In this work, we developed two methods, CSAlign and CSAlign-Dock, which predict ligand and protein–ligand complex structures, respectively, utilizing known structure information on a related ligand. CSAlign performs compound-to-compound alignment considering full ligand flexibility using CSA global optimization. Compared to other compound structure alignment methods, CSAlign shows near-perfect self-alignment performance.

CSAlign-Dock can be viewed as an alignment-based protein–ligand docking method that uses the information of the template complex structure and the docking score to better predict complex structure. The performance of CSAlign-Dock tested on a cross-docking benchmark showed that it successfully combined the information-based alignment and *ab initio* docking. CSAlign-Dock significantly outperforms other *ab initio* protein–ligand docking methods in the cross-docking setting, where slight conformational changes in protein structures often lead to poor predictions in conventional docking.

However, the absolute success rate of CSAlign-Dock is still far from perfect, and the method cannot deal with large protein conformational changes. It is still expected that CSAlign-Dock can be applied in various practical cases where reference complex structures are available, in many hit discovery and lead optimization applications. The predicted structure can also be used as a starting point for further affinity or binding free energy calculation, which is expected to rely highly on the quality of the input structure. The web server for CSAlign and CSAlign-Dock is also freely available.

## CRediT authorship contribution statement

**Sohee Kwon:** Conceptualization, Methodology, Data curation, Software, Formal analysis, Validation, Writing – original draft. **Chaok Seok:** Conceptualization, Supervision, Writing – review & editing, Funding acquisition.

## Declaration of Competing Interest

The authors declare that they have no known competing financial interests or personal relationships that could have appeared to influence the work reported in this paper.
