# Supplementary material for: CSAlign and CSAlign-Dock: Structure alignment of ligands considering full flexibility and application to protein–ligand docking
Source: Comput Struct Biotechnol J. 2022 Nov 26;21:1–10. doi: 10.1016/j.csbj.2022.11.047 (PMC9719078; doi:10.1016/j.csbj.2022.11.047)
Supplement: Supplementary data 1 [file mmc1.docx]

**Supplementary Information**

CSAlign and CSAlign-Dock: Structure alignment of ligands considering full flexibility and application to protein-ligand docking

Sohee Kwon and Chaok Seok^[[1]](#footnote-1)^*

Department of Chemistry, Seoul National University, Seoul 08826, Republic of Korea

and Galux Inc, Seoul 08738, Republic of Korea

**Supplementary Figures**


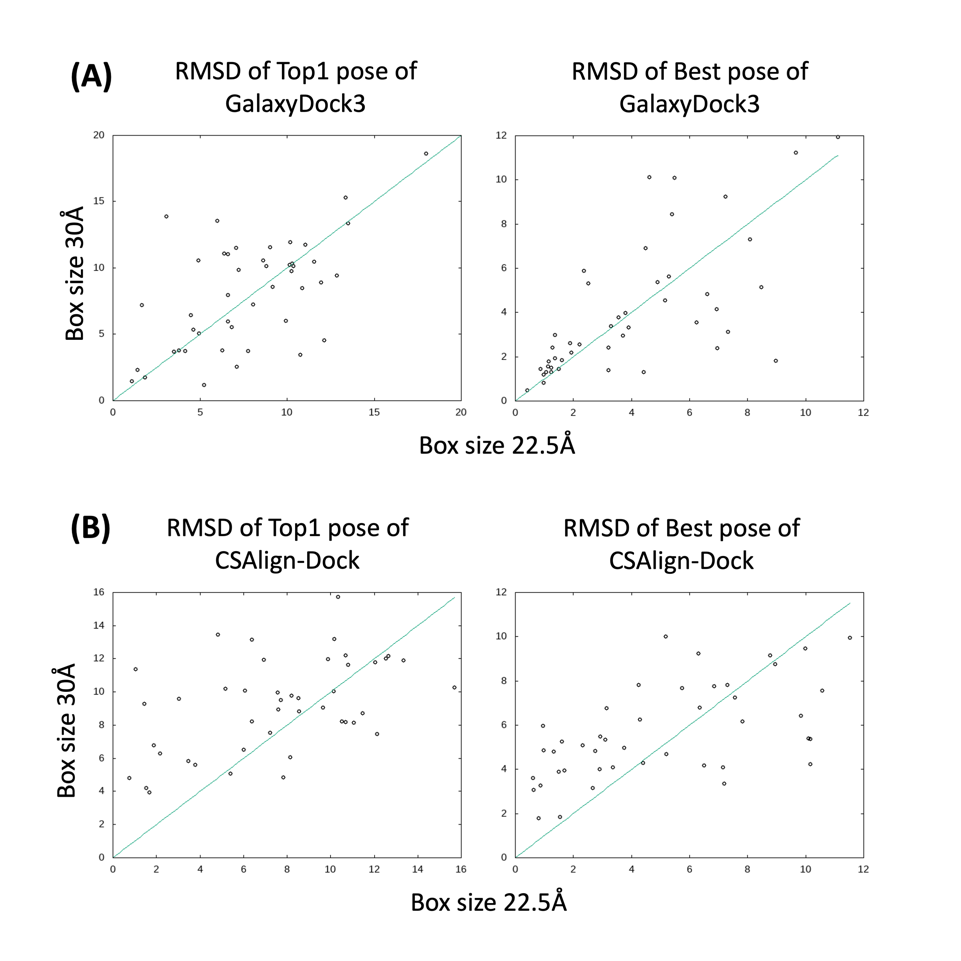


**SF 1.** Comparison of docking results for 45 large query ligands using the default (cubic box size of 22.5 Å) and large (30 Å) docking boxes for GalaxyDock3 (A) and CSAlign-Dock (B). The average number of heavy atoms (torsions) for the 45 selected ligands is 37 (10) compared to 23 (5) and for all ligands. The large conformational search space for the large boxes and the large ligands makes docking more difficult.

(A)

(B)

**SF 2.** (A) Dependence of success rates (with the RMSD cutoff of 2.5 Å) on 3D compound similarity measured by the alignment score for CSAlign, CSAlign-Dock, and GalaxyDock3. The 3D alignment score was measured after the best rigid alignment of each ligand to the corresponding crystal reference ligand structure. The yellow bar shows the success rate of the top 1 pose and the blue bar the best pose. The low success rates for low 3D alignment scores of < 0.2 is related to the very small number of ligands in the range (3 and 4 for the range of 0 to 0.1 and of 0.1 to 0.2, respectively) shown in the middle figure of (B) compared to the left figure which shows 2D similarity. The scatter plot on the right panel of (B) shows that 2D and 3D similarities are not highly correlated.

**Supplementary Tables**

**ST 1.** PDB IDs for the benchmark set consisting of 477 query compounds (and the corresponding proteins to bind) and 1,724 query-reference pairs of protein-ligand complexes.

| Query | Reference |
| --- | --- |
| 5xva | 2x4z,4app,4o0x,4o0y,5xvg |
| 4i5c | 4e4l,4e4n,4e5w,4ei4,4ivb,4ivc,4k77 |
| 6gl9 | 5lwm,6gla,6glb |
| 1txr | 1ft7,1igb,3b3s,3vh9 |
| 1pxn | 1pxo,1pxp,6gue |
| 2c97 | 2c92,2c94 |
| 2wgj | 3zbx,3zc5,3zxz,4aoi,4ap7 |
| 4j7d | 4j7e |
| 3f7h | 2i3h,2i3i,3f7i,3gt9,3gta,3uw5 |
| 5ovv | 5ovc,5ovp |
| 6ce6 | 6ced |
| 3ov1 | 3ove,3s8l,3s8n |
| 5mgj | 5mgk,6fgg |
| 1det | 6rnt |
| 1lkk | 1bhf |
| 5f0f | 5eyr,5f08,5ioz,5j1r,5j3l |
| 3pb9 | 3pb7,3pb8 |
| 1fh7 | 1fh8,1fh9,1fhd,1j01 |
| 2bet | 2bes |
| 4b6r | 2xd9,4b6s |
| 3b4p | 3jup |
| 3mfw | 3f80,3kv2,3lp4,3lp7,3mfv,3mjl |
| 2fqw | 2fqx,2fqy |
| 5lvd | 5js3,5jt9,5lif,5lwd,5m9w,5ma7,5mnr,5n2t,5n2z,5n31,5n34,5n3v,5n3y |
| 6ckw | 6b7a,6chp |
| 2amt | 2gzl |
| 5f1x | 3ldp,5evz,5exw,5ey4,5f2r |
| 4ks4 | 4ks1 |
| 4a95 | 5g1z |
| 4do5 | 4do4 |
| 4ua8 | 4uac |
| 4gzx | 4gzp,4gzt,4gzw |
| 2qwe | 1f8b,1f8c,1f8d,1f8e,2qwb,2qwf |
| 4i72 | 4i71,4i74 |
| 5t8p | 5t8o |
| 1fq5 | 2jxr |
| 5l9i | 5l9g |
| 6bm5 | 6bdy |
| 4mhz | 4mhy |
| 2hhn | 2f1g |
| 5g5f | 5kej |
| 1ql9 | 1j14 |
| 5aba | 2vuk,4agl,4agm,4agn,4ago,4agp,4agq,5a7b,5aoi,5aoj,5aol,5g4o,5o1d,5o1f,5o1h |
| 4cr5 | 4cr9,4cra,4crb,4crc,4crf |
| 5fbi | 5fck,6ftz,6fuh,6fui,6fuj |
| 1s19 | 1ie9 |
| 3b3x | 1i2s |
| 5ito | 4pow,4pox,5ot9,5ota |
| 3bxe | 3bxf,3bxg |
| 1jak | 1hp5,5fcz |
| 1ogg | 1ur9 |
| 2jg0 | 2jjb |
| 4qfp | 4qfl,4qfn,4qfo |
| 3f3d | 3f3c,3f3e,3f48 |
| 4n6g | 4n5d,4n7m,4nbk,4nbl,4nbn |
| 1e6q | 1e6s |
| 2vmd | 2vmc |
| 3suw | 3sur,3sus,3sut,3suv |
| 3uxk | 3uxl,4fp1,4m6u |
| 5g1a | 5g17 |
| 2wvz | 2wzs |
| 1ajp | 1ai4,1ai5,1ai7,1ajn,1ajq |
| 1qbq | 1o1s |
| 3t6b | 5e3a |
| 5kby | 1n1m,2oag,2ole,4jh0,4lko |
| 187l | 184l,185l,186l,188l,1l83 |
| 1zs0 | 1jao,1jaq,3tt4 |
| 1lke | 1lnm |
| 4k7n | 4k7i,4k7o,4mmm |
| 4km0 | 4km2 |
| 3i51 | 3i4y |
| 1hn4 | 3qlm |
| 2q88 | 2q89 |
| 3hmo | 3hmp |
| 3nht | 3nhi |
| 2ves | 4lch,5drr |
| 4clj | 2yfx,4cd0,4cmo,5kz0 |
| 5wbo | 5wbm |
| 5vcy | 5vcv,5vcw,5vcz,5vd0,5vd1,5vd3 |
| 4ytc | 4f09,4gfm,4iva,4jia,4yth,5l3a |
| 3zll | 4arw |
| 1g98 | 1koj |
| 3uil | 3umq |
| 1jcx | 3.00E+12 |
| 1n3i | 1g2o |
| 2yb0 | 2yay,2yaz |
| 3ocp | 4z07 |
| 3kr4 | 4k3n,4r76,5cbm |
| 4dv8 | 1yqy |
| 1b4h | 1b05,1b0h,1b1h,1b2h,1b32,1b3f,1b3g,1b3h,1b3l,1b40,1b46,1b4z,1b51,1b58,1b5h,1b5i,1b5j,1jeu,1jev,1qka,2rkm |
| 4h75 | 5jsj |
| 4avs | 2w08,3kqr |
| 4kn0 | 4kmz |
| 1kug | 1kui,1kuk |
| 1jqy | 1pzi |
| 1mrn | 1mrs |
| 4ew2 | 1njs |
| 2evl | 2euk |
| 4deu | 1bm7,1e4h,3cfn,3cft,3kgu,3nee,3neo,3p3r,3p3s,3p3t,4der,4des,4det,4dew,5ayt,5boj,5en3,5ihh,5l4i,5l4j,5l4m,5u49,5u4b,5u4d |
| 4zzx | 4zzy |
| 1fkw | 1add |
| 4kyk | 4kyh |
| 3l3l | 2rd6,3gjw,3l3m,4und |
| 3zm9 | 4br3,4cg8,4cg9,4cga,4da5,5afv,5eqe,5eqp,5eqy,5ftg,5fut |
| 2qta | 1rp7 |
| 1rd4 | 3f78 |
| 2j7f | 2cet,2j77,2j79,2j7b,2j7d,2j7e,2j7g |
| 5meh | 4ayp,4ayq,5ne5 |
| 2zx6 | 2zwz,2zx7,2zx8,2zxd |
| 1ec9 | 1ecq |
| 1u71 | 4g95 |
| 2bfr | 2bfq |
| 5aan | 6epa |
| 2buv | 1eoc |
| 2zz1 | 1x1z,2zz2,3g1d,3g1v,3wjw |
| 4luz | 4o0a,4r4c,4r4i,4r4o,5e7n |
| 1o0h | 1afk,1afl,1jn4,1jvu,1o0f,1o0m,1o0n,1qhc,1rnm,1rpf,1u1b,1w4p,1w4q,1z6s,2w5g,2xog,3d6o,3d6p,3d8z,4g8v,4g8y,4g90 |
| 1ogx | 1e3v,5g2g |
| 5dit | 4jfk,4jfm,4w9o |
| 5y94 | 5cy9,5dx4 |
| 3oyw | 3oy8 |
| 4hp0 | 4tun |
| 5a81 | 4qsu,4qsv,4tt2,4tte,4tu4,4tz2,5a5q |
| 1q54 | 2vnp |
| 3rlb | 4pop |
| 6ayq | 6ayo,6ayr |
| 4wko | 4ffs,4wkn,4wkp,4ynb |
| 2v59 | 2v58,3rv4 |
| 1ew8 | 1ew9 |
| 3t3u | 3sxf |
| 3hl8 | 3hp9 |
| 4hfp | 3bv9 |
| 3fj7 | 3fjg |
| 4pcs | 2wvt,2xib,2xii,4j28,4jfs,4pee |
| 2pqc | 2pqb |
| 1o5e | 1p57 |
| 3cz1 | 3cyz |
| 3rf4 | 3rf5 |
| 4isi | 2b7d,2bz6,2flr,4ish,4na9,4x8u,4x8v,5l30,5u6j |
| 2vot | 2vjx,2vl4,2vmf,2vo5,2vqt |
| 4iif | 4iic,4iid,4iie |
| 3qfy | 3qfz |
| 3cct | 3ccw,3cd0,3cd5,3cdb |
| 4cu7 | 4cu8 |
| 5nn5 | 5nn6 |
| 4ido | 4idn |
| 3ebp | 3ebo |
| 4xu3 | 3rux,4xtv,4xtw,4xtx,4xty,4xtz,4xu0,4xu1,4xu2 |
| 3uo4 | 3uod,3up2 |
| 2oi0 | 3b92,3ewj,3kmc,3lea |
| 5evd | 2fu8,2qdt,5dpx,5evb |
| 5efa | 5efc |
| 4a4v | 2yfe,3b1m,3u9q,4a4w,5lsg |
| 4few | 4gkh,4gki |
| 1sln | 1b8y,1ciz,2d1o |
| 5ia0 | 5ia1,5nki,6fnf,6fng |
| 4rrg | 4rrf |
| 3hb4 | 1i5r |
| 2zcr | 2zcq,3acw,3acx,3tfn,3tfp,3vje,4e9u,4ea2 |
| 3iod | 3coy,4muf |
| 2c3i | 1xws,2xj1,2xj2,3bgq,3jy0,3jya,4k0y,4k18,4n6z,5dwr,5ipj,5v82 |
| 5oha | 5oh2,5oh3,5oh4,5oh7,5oh9 |
| 5ufp | 5tbm |
| 5lvr | 5fe6,5fe7,5fe9,5lvq |
| 5ueu | 5uez,5uf0 |
| 3ikd | 3ikg |
| 5f2p | 4xy8,5eu1,5f1h,5f25,5i7y,5igm,5ji8 |
| 2i2c | 4dy6 |
| 6eqp | 6ep4 |
| 5ceq | 5cep,5vo1 |
| 2v2v | 2v2q |
| 4gu6 | 4k9y |
| 2wly | 2wlz,2wm0 |
| 2pwd | 2pwg |
| 1wur | 1wuq |
| 3c2o | 3c2f,3c2r |
| 4m0f | 4m0e |
| 5bwc | 1e66,1gpk,1gpn,1h22,3zv7,5nap,5nau |
| 3iae | 3d7k |
| 5laq | 1ro6,3o56 |
| 6b96 | 6b97,6b98 |
| 2ojg | 2ojj,4qyy |
| 3ip5 | 3ip6,3ip9 |
| 5lyr | 4ad2,4ad3,5m17,5m5d,6hmg |
| 1usi | 1usk |
| 3vw1 | 3vvy,3vw2 |
| 6eij | 6eif,6eiq,6eir,6eis |
| 4wop | 6cvf,6cvv,6czb,6czc,6cze |
| 5kly | 5i2e,5ipc,5kma,5wa8 |
| 4zv1 | 4zv2 |
| 4ygf | 4yha,5tt3,5tuo |
| 1qkt | 2ayr |
| 2a5b | 2a5c,2a8g,5chk |
| 1kdk | 1lhu |
| 5we9 | 5w44 |
| 5h1t | 5h1v |
| 5cp9 | 5cs3,5ct2 |
| 5ldm | 5ldp |
| 2w8y | 1a28,1sr7 |
| 4b5t | 4b5s |
| 5fl4 | 5fl5,5fl6 |
| 2vxn | 1amk |
| 3g0w | 1e3g,1i37,2ihq,3b5r,3b65,3b66,3b67,3b68 |
| 4tim | 1iih,1kv5,1trd |
| 4l51 | 4l4z,4l50 |
| 5l7h | 5l7g |
| 4hws | 4hwo,4hwp |
| 3u8k | 1uw6,3u8j |
| 4azc | 4azg |
| 2c80 | 1oe8 |
| 10gs | 2gss,3gss,5j41 |
| 4zbf | 4hw3,4zbi |
| 6g34 | 6g35,6g36,6g37,6g38 |
| 6hqy | 6htg |
| 4c1u | 4c1t |
| 3qps | 3qqa |
| 4cpz | 4cpy |
| 1w9v | 1w9u,2iuz |
| 1qy1 | 1qy2 |
| 5mkr | 5aqz,5mks,6fhk |
| 3k00 | 3jzj |
| 4mre | 4mrg,4np2,4np3 |
| 5anv | 4c9x,5ant,5anu,5fsn,5fso,6f20 |
| 4f7v | 1ex8 |
| 5iyy | 5ijr,5j1x,5jgi,5jgq,5jhk,6fmc |
| 5upe | 4n9c,5upf |
| 3i3b | 1px4 |
| 4kx8 | 4kxb |
| 3ebi | 3ebh,3q44,3t8v,4k5p,4r5t,4zw5,4zw6,4zw7,4zw8,4zx3,4zx4,6eed |
| 3l4v | 3ctt,3l4u,3l4w,3l4z |
| 4o2b | 5yl2 |
| 4dhl | 4dsy |
| 1m0o | 1m0n,1m0q,1zc9 |
| 5f2u | 5e8f,5x74 |
| 2vj8 | 2r59,3b7r,3b7u,3fh7,4l2l |
| 3ps1 | 3p3g |
| 3zsx | 3zso,3zsq,3zsy,3zt2,3zt3,4ceb,4cgi,4cj4,4cjp,4cjq,4cjr,4ck3 |
| 4rlt | 4rlu,4rlw |
| 5gja | 5gj9 |
| 2h4k | 1bzc,1bzj,1c83,1c84,1c87,1c88,1ecv,1g7f,1g7g,1kav,1l8g,1nl9,1nny,1no6,1nz7,1ony,1onz,1pyn,1qxk,1xbo,2azr,2b07,2f6t,2h4g,2hb1,2nt7,2nta,2qbp,2qbq,2qbr,2qbs,2zmm,2zn7,3eax,3eb1,4i8n,5kab,5t19 |
| 5oku | 5oei |
| 3ckz | 3cl0 |
| 2xn5 | 2xn3 |
| 3rm4 | 3rm9 |
| 4q4o | 1enu,1f3e,1k4g,1k4h,1q65,3gc5,3rr4,4lbu,4leq,4pum,5jxq |
| 5lz5 | 5lz4 |
| 5om7 | 5om2 |
| 3ahn | 3aho |
| 1pbq | 1pb8,1pb9,1y1z,2a5s,5dex,5vih,5vij |
| 3bgs | 1rr6,3k8q,5etj |
| 3arw | 3arp,3arq,3arx |
| 6f9u | 1o86,2oc2,3bkk,3bkl,3l3n |
| 3dri | 3drf,3drg |
| 3cj4 | 3cj5,4eo6,4eo8 |
| 5wyz | 5wyx |
| 5ha1 | 5hbs |
| 5nvx | 4bkt,4w9c,4w9d,4w9f,4w9h,4w9k,5lli,5nvv,5nvw,5nw1,5nw2,6fmj,6gfz |
| 6ht1 | 6hpw |
| 4lxd | 4ieh,4lvt |
| 3zln | 3qkd |
| 4g0y | 4g0p,4g0q,4g0z |
| 1ugx | 1ws4 |
| 1oxr | 1td7 |
| 3s0d | 3s0b,3s0e |
| 4qf8 | 1fv0,1jq8,1kpm,1q7a,1sv3,2arm,3h1x,4qem,4qer,4qf7,4qgd |
| 4lk7 | 2wyf,3zyf,4a6s,4ljh,5d21 |
| 5tb6 | 5ep7,5mme,5mpn |
| 4nra | 4rvr,5e73,5e74,5mge,5mgf |
| 4jkw | 4k55 |
| 4uye | 5eq1,5etb,5mwh,5o5a,5ov8,6ekq |
| 5fh7 | 5fh8,5hrv,5hrw,5hrx,5ii2 |
| 5mro | 5mrm |
| 1bgq | 1amw,2cgf,2fxs,2vw5,2vwc,2weq,2yge |
| 3h8b | 3h89 |
| 4k6i | 3ozj,4k4j,4m8e,4poh,4poj,4pp3,4pp5 |
| 4.Oct | 4o61 |
| 1fkb | 1d7i,1d7j,1fkf,1fkg,1fkh,1fki,1j4r |
| 1km3 | 1loq |
| 5tmp | 4tmk |
| 4iue | 3kr8,4j21,4j22,4j3l,4kzq,4kzu |
| 3c56 | 3c52,3n9r |
| 6g2m | 1q91,6g2l |
| 4re4 | 4re2 |
| 2hjb | 2q7q |
| 1a69 | 1k9s |
| 6eqx | 4omc,4ryd,6eqv,6eqw |
| 3f5j | 3f5l |
| 2ya7 | 2ya6,2ya8 |
| 6d56 | 6d55,6d5e,6d5g,6d5h,6d5j |
| 1odj | 1odi |
| 1y6q | 1nc1,1nc3,1y6r |
| 3g34 | 3g2y,3g2z,3g30,3g32,3g35,4de0,4de1,4de2 |
| 6h36 | 3ml5,6h37,6h38 |
| 4qj0 | 4ht2,4kp5,4kp8,4qjw,5llo,5llp,5msb |
| 4m13 | 4m12 |
| 4lrr | 1f4e |
| 4o3c | 1p1n,1p1o,1p1q,1syh,1wvj,1xhy,2al5,3bft,3tza,4g8m,4igt,5cbs,5nih |
| 6h33 | 1avn,1bcd,1bn1,1bn3,1bn4,1bnn,1bnq,1bnt,1bnu,1bnv,1bnw,1cnw,1cnx,1cny,1g1d,1g46,1g48,1g4o,1g54,1i9n,1i9p,1if7,1okl,1ttm,1xpz,1xq0,1yda,1z9y,1zfq,1zge,2ez7,2h15,2hl4,2hnc,2hoc,2o4z,2pou,2pov,2pow,2q1q,2q38,2weg,2weh,2wej,2weo,2x7t,2x7u,3b4f,3bl0,3bl1,3d8w,3d9z,3daz,3dd0,3dd8,3eft,3f8e,3ffp,3hkn,3hkq,3hkt,3hku,3ibi,3igp,3k2f,3kwa,3m1k,3m3x,3m40,3m5e,3m67,3m96,3mhc,3mhi,3mhl,3mhm,3mho,3ml2,3mmf,3mna,3myq,3mzc,3n0n,3n2p,3n3j,3n4b,3nb5,3ni5,3oil,3oku,3oy0,3oyq,3p4v,3p58,3p5l,3po6,3r16,3r17,3ryj,3ryv,3ryx,3ryy,3ryz,3rz0,3rz1,3rz5,3rz7,3rz8,3s71,3s72,3s73,3s76,3s77,3s78,3t5u,3t82,3t83,3t84,3v7x,3vbd,4bf1,4bf6,4e3g,4ht0,4itp,4iwz,4jss,4kni,4knj,4m2r,4m2u,4mo8,4q6d,4q6e,4q7p,4q7s,4q7v,4q7w,4q81,4q83,4q87,4q8x,4q8y,4q90,4q99,4q9y,4qtl,4r5b,4rfc,4rfd,4rn4,4rux,4ruz,4yxi,4yyt,4z0q,4z1e,4z1j,4z1k,4zx0,5amd,5amg,5aml,5byi,5e28,5e2k,5e2r,5e2s,5eh5,5eh7,5eh8,5ehv,5ehw,5eij,5ekm,5fdc,5fdi,5flo,5flq,5fls,5flt,5fng,5gmn,5j8z,5ljq,5ljt,5llc,5lle,5llg,5llh,5mjn,5n0d,5n1r,5n1s,5n24,5n25,5nea,5nee,5nxg,5nxi,5nxo,5nxp,5ny1,5ny3,5o07,5sz0,5sz1,5sz3,5tfx,5ti0,5txy,5ty9,5tya,5u0d,5u0e,5u0f,5u0g,5uln,5vgy,5wex,6b4d,6b59,6c7w,6c7x,6ebe,6ecz,6equ,6g3q,6got,6h29,6h2z,6h34 |
| 5oq8 | 5oot,5op5 |
| 5za7 | 1ejn,1f5k,1f5l,1w0z,1w11,1w13,2r2w,3kgp,3mhw,4h42,5za8,5za9,5zae,5zaf,5zag,5zaj,5zc5 |
| 1v0k | 1od8,1v0l |
| 1ik4 | 1egh |
| 6dar | 4ql1,5m23,5m25,5sxm,6d9x,6dai,6dak |
| 5ive | 5ivv |
| 1w3l | 1e5j,1h5v,1ocq,1w3k,8a3h |
| 5cau | 4rj8,5c8n,5cap,5caq,5hcy |
| 3ed0 | 3b7j,3cf8 |
| 4u73 | 4u1b,4u69,4u6c,4u70 |
| 1oar | 1oau |
| 6gnm | 6gnp,6gnr,6gnw,6gon |
| 2b1g | 2b1i |
| 4xmb | 5x54 |
| 3pyy | 3k5v,6hd6 |
| 3n7a | 2xb8,3n76,3n86,3n8k,4b6p,4ciw,4kiu |
| 2uy3 | 2uy4 |
| 4gue | 3ubd |
| 4rqk | 4rqv |
| 6h2t | 6h1u |
| 2hmv | 2hmu |
| 3hfb | 3hf8 |
| 4omk | 4omj |
| 5u14 | 5u0w,5u0y,5u0z,5u11,5u13,5v79,5v7a |
| 5dq8 | 5dqe |
| 4jx9 | 4jwk |
| 3i7e | 1kzk,3aid,3el1 |
| 2h3e | 1d09,2fzc,2fzg,2fzk |
| 5l8y | 5g2b,5g57,5g5v,5l8c |
| 1x38 | 1x39 |
| 2x97 | 2x8z,2x91,2x95,2x96,2xhm |
| 4dkq | 4dko,4dkp,4i54 |
| 1ctu | 1ctt |
| 4q3u | 4q3t |
| 3iw6 | 3gcp,3iw5 |
| 3u90 | 3f33,3f37 |
| 4je8 | 4je7 |
| 3str | 3sw8 |
| 4yzu | 4z0k,4zae,5egm |
| 4b3b | 5fou |
| 4o9v | 2gv6,2gv7,4jyt,4jz1,4jzi |
| 2gkl | 3iof,3iog |
| 3sm2 | 3slz |
| 5j7q | 4wrb,5hvs,5hvt,6b1k,6cbf |
| 2boj | 2jdp,3zdv |
| 2e2r | 2p7a,2p7z |
| 2y7x | 1ezq,1f0r,1f0s,1fjs,1ksn,1lpg,1lpk,1lpz,1mq5,1mq6,1nfu,1nfw,1nfx,1nfy,1xka,1z6e,2boh,2bq7,2j34,2j4i,2j94,2j95,2jkh,2p16,2p95,2ra0,2uwl,2uwo,2uwp,2vh0,2vh6,2vvc,2vvu,2vvv,2vwl,2vwm,2vwn,2vwo,2w26,2wyj,2xbv,2xbw,2xbx,2xc0,2xc4,2y5f,2y5g,2y5h,2y7z,2y81,3cs7,3ffg,3liw,3m36,3m37,4a7i,4y79,5k0h |
| 3tvc | 2d1n,3kek,456c,4l19 |
| 1mmq | 1mmr |
| 4elg | 4elf |
| 5ewy | 5ewk |
| 4y59 | 4y5d,5b5f |
| 5cst | 4emf,4emr,4zt8,5cso |
| 4ibe | 4ibb,4ibc,4ibd,4ibf,4ibg,4ibi,4ibj,4ibk |
| 2xxr | 2xxt |
| 4ykj | 4ykk |
| 3uz5 | 3uzj |
| 3c88 | 3c8a,3c89,3qw5 |
| 4u5o | 4u54,4u5n,4u5s |
| 4afg | 4b5d |
| 3ddf | 1ps3,2f7o,2f7p,3d4y,3d50,3d51,3d52,3ddg,3dx1,3dx2,3dx4,3ejp,3ejq |
| 4ad6 | 3qbc |
| 3f16 | 1rmz,2hu6,3ehx,3ehy,3f15,3f17,3f18,3f19,3f1a,3lir,3ljg,3lk8,3lka,3n2u,3n2v,3nx7,3ts4,3tsk,4efs,4gql,4gr0,4gr3,4gr8,5cxa,5d3c |
| 4x5p | 1uwf,4att,4auj,4av4,4av5,4avh,4avi,4avj,4buq,4cst,4lov,4x50,4x5q,4x5r |
| 4gny | 3uev |
| 4gj3 | 4gih,4gii,4gj2,5wal |
| 6g0z | 6g14 |
| 2reg | 2rin |
| 1lgt | 1kmy |
| 4cd4 | 4cd5 |
| 5gsa | 3jzh,5k0m |
| 3znr | 3zns |
| 6ej3 | 5i3v |
| 5ix0 | 4lxz,4ly1,5iwg |
| 4ovf | 4ovg,4ovh,4pnu |
| 1stc | 1q8t,1q8u,1q8w,1xh4,1xh5,1ydr,1yds,1ydt,2jds,3agl,3dnd,3dne,4uj1,4uj2,4uja,5izf |
| 4r73 | 4r74,4r75 |
| 4nkt | 4nku |
| 2x0y | 2cbj,2j62,2xpk |
| 1ppl | 1apv,1bxo,1bxq,1ppk,1ppm |
| 3gs6 | 3gsm |
| 3pbb | 2afw,2afx |
| 4er2 | 1epo,4er1,5er1,5er2 |
| 6fnr | 4pin |
| 5y13 | 2hnx,5d45,5d47,5d48,5edb,5edc,5y12 |
| 1hi5 | 1hi3,1hi4,5e13 |
| 1vyf | 1vyg |
| 4tjz | 1hmr,1hms,5hz9 |
| 5i1q | 5i29 |
| 3t1m | 1kjr,5e89,5h9r,6eog,6eol |
| 4f6u | 4f6w |
| 4d4d | 5m77,5n0f |
| 4nxu | 4nxv |
| 1lf2 | 1lee |
| 4aje | 4aji |
| 4ek9 | 3qox,3sr4 |
| 6hh5 | 6hh3 |
| 2rk8 | 2rkd,2rke,3mof |
| 4tln | 1os0,1qf0,1qf1,1qf2,1tlp,1tmn,1z9g,1zdp,2tmn,3fcq,4tmn,5tmn |
| 2wed | 2web,2wec |
| 1nja | 1bp0 |
| 4ufl | 4ufh,4ufi,4ufj,4ufk,4ufm |
| 2vw1 | 2vw2 |
| 6eq8 | 6epz,6eq1 |
| 2wca | 2j4g,2jiw,2vvn,2w4x,2w66,2w67,2xj7,2xm1,2xm2 |
| 2cle | 2clh,2cli |
| 5uc4 | 5ucj |
| 3oe4 | 3hvi,3hvj,3oe5,3ozr,3ozs,3ozt,3u81,5k03 |
| 5ev8 | 5ewa |
| 6h5x | 2xyd,3nxq,6en5,6f9v |
| 4lar | 4gqp |
| 2wf5 | 1z4o |
| 1utm | 1utj,1utl |
| 1t31 | 3n7o |
| 4.00E+67 | 4o6w |
| 4zeb | 4ra1,4zec,4zei,4zek |
| 1y3n | 1y3p |
| 1q1g | 1nw4 |
| 1uwt | 1uwu,2ceq |
| 4kwf | 4kwg |
| 1u33 | 1xd0,3old,4gqr |
| 2qtg | 2qtt,3lgs |
| 4a6l | 2za5 |
| 1lcp | 1lan,2ewb |
| 4rpo | 4rpn |
| 5vsf | 5tuz |
| 5vc3 | 5vc4,5vd2 |
| 4knn | 3czv,4hu1,4knm,4qjx |
| 2nn1 | 1azm,2nmx,2nn7,3lxe,6evr,6ex1,6f3b,6faf,6g3v |
| 6cn5 | 5g45,5ufr,5vb5,5vb6,5vb7 |
| 2vpo | 2vpn |
| 3vhk | 4ag8,4asd |
| 3b50 | 2cex |
| 4h85 | 4dzy,4h7q |
| 2ctc | 1f57,1hdq,2rfh,3fvl,3fx6,3kgq |
| 2ovy | 2o8h,2ovv |
| 6upj | 1ivp,5upj |
| 3c4h | 3fhb |
| 5k1f | 5k1d |
| 5ef7 | 5eei,5eek,5een,5efh,5efj,6csp,6csq,6csr,6css |
| 3gr2 | 1xgi,2pu2,2r9w,2r9x,3gqz,3gtc,3gv9,3gvb,4jxs,4kz3,4kz4,4kz6,4kz7 |
| 3i4b | 1q5k,4acc |
| 4hym | 4hy1 |
| 4rra | 4rr6 |
| 3ppq | 3ppp,3ppr |
| 4j48 | 4j44,4j45,4j46,4j47 |
| 4mn3 | 4x3k |
| 4az5 | 4az6 |
| 2cc7 | 2ccb,2ccc |
| 5oa2 | 5o9o |
| 2ptz | 2pu1 |
| 3pcj | 3pce,3pcf,3pcg,3pck,3pcn |
| 1x8r | 2pq9 |
| 2byr | 2wn9,2wnc,2x00,3c79,4xk9 |
| 1yej | 1yei |
| 3sue | 3su4 |
| 2wos | 2wor |
| 5ta2 | 5lud,5t9u,5t9w,5t9z,5ta4,6gji,6gjj,6gjl,6gjm,6gjn,6gjr |
| 3a1d | 3a1c |
| 3d91 | 2iko,3own,4s1g |
| 2f9k | 1yq7,2f94,4pvx,4pvy |
| 4umb | 4uma |
| 1r1j | 1r1h |
| 4oc0 | 2xef,2xei,2xej,3iww,3rbu,3sjf,4ngm,4ngn,4ngp,4oc2,4oc3,4oc5 |
| 2jkp | 2jke,2zq0 |
| 6ma3 | 6ma2,6ma4 |
| 6std | 2std,3std,4std,5std |
| 3ozp | 3nsn,3wmc |
| 5fsx | 5fsy |
| 1fd0 | 1fcx,1fcy,1fcz |
| 4pf5 | 4czs |
| 1lag | 1laf,1lah,1lst |
| 1eld | 1bma,1ela,1elb,1elc,1ele |
| 4io3 | 4io2,4io4,4io5,4io6,4io7 |
| 1n4h | 1nq7 |
| 5umx | 6bbx |
| 2pvu | 2q2a |
| 5l9o | 5lom |
| 5qay | 5qa8,5qal |

**ST 2.** PDB IDs for the CDK2 benchmark set.

| 1h1q |
| --- |
| 1h1r |
| 1h1s |
| 1ogu |
| 1oi9 |
| 1oiu |
| 1oiy |
| 2c6k |
| 2c6m |
| 2g9x |

1. * Correspondence to: Chaok Seok, Phone: +82-2-880-9197, E-mail: [chaok@snu.ac.kr](mailto:chaok@snu.ac.kr) [↑](#footnote-ref-1)
